# Antibiotic and Disinfectant Susceptibility Patterns of Bacteria Isolated from Farmed Fish in Kirinyaga County, Kenya

**DOI:** 10.1155/2020/8897338

**Published:** 2020-07-30

**Authors:** Daniel W. Wanja, Paul G. Mbuthia, Robert M. Waruiru, Lilly C. Bebora, Helena A. Ngowi, Philip N. Nyaga

**Affiliations:** ^1^University of Nairobi, College of Agriculture and Veterinary Sciences, Department of Veterinary Pathology, Microbiology and Parasitology, P.O. Box 29053-00625, Kangemi, Nairobi, Kenya; ^2^Animal Health and Industrial Training Institute (AHITI) Kabete, P.O. Box 29040-00625, Kangemi, Nairobi, Kenya; ^3^Sokoine University of Agriculture, College of Veterinary and Medical Sciences, P.O. Box 3000, Chuo Kikuu, Morogoro, Tanzania

## Abstract

Fish bacterial pathogens cause diseases which result in a considerable economic impact on the aquaculture industry, necessitating the use of antimicrobials for their control. However, intensive and indiscriminate use of antimicrobials has led to increased occurrence of drug resistance in pathogenic bacteria, as well as normal flora. The aim of the current study was to determine the susceptibility patterns of bacteria isolated from fish, with respect to some commonly used antibiotics and disinfectants. Bacteria were isolated between December 2017 and April 2018 from farmed Nile tilapia, African catfish, goldfish, and koi carp in Kirinyaga County, Kenya. Antibiotic and disinfectant susceptibility patterns of 48 isolates belonging to the genera *Aeromonas*, *Proteus*, *Klebsiella*, *Citrobacter*, *Salmonella*, *Streptococcus*, *Pseudomonas*, *Escherichia*, *Serratia*, and *Micrococcus* were established using the Kirby–Bauer disc diffusion method and agar well diffusion technique, respectively. The antibiotics evaluated included ampicillin, tetracycline, co-trimoxazole, streptomycin, kanamycin, gentamicin, co-trimoxazole, and chloramphenicol, while the disinfectants tested were quaternary ammonium compound, formalin, hydrogen peroxide, sodium hypochlorite, and iodine. All the bacteria except *Micrococcus*, *Escherichia*, and *Salmonella* species showed multiple drug resistance patterns. *Streptococcus* showed resistance to six antibiotics, while *Proteus*, *Pseudomonas*, and *Serratia* were resistant to five antibiotics. The multiple antibiotic resistance index ranged from 0.1 to 0.8, with *Streptococcus* spp. having the highest score value. All the organisms were sensitive to gentamicin, while co-trimoxazole and ampicillin showed the highest resistance at 73% (*n* = 34) and 62% (*n* = 31), respectively. Most of the disinfectants showed antibacterial activity with varying magnitudes. The isolates were 100% sensitive to hydrogen peroxide and formalin, but were resistant to sodium hypochlorite at recommended user-dilution. The study has shown that some of the bacterial isolates were resistant to common antibiotics and disinfectants; thus, it is recommended to include an antibiogram whenever making any therapeutic decision. The resistant bacteria may transmit resistance genes to other fish bacteria and also to human bacteria, thus making it difficult to treat the resultant disease(s); thus, there is a possibility that these resistant bacteria may be transmitted to humans who consume or handle the carrier fish. It is, therefore, advisable that fish are cooked properly before consumption, so as to kill bacteria that may be present.

## 1. Introduction

A wide range of fish bacteria have the potential to cause diseases, making them one of the most important concerns for the aquaculture sector. So far, many pathogenic microorganisms, such as *Aeromonas hydrophila*, *Pseudomonas* spp., *Citrobacter freundii*, *Streptococcus iniae*, *Flavobacterium columnare*, and *Edwardsiella tarda*, among others, have been reported as aetiological agents of fish diseases [1]. Disease outbreaks can, in turn, bear negative implications on the quality and rate of production, worse being fish mortality. Thus, based on this aspect and the increased disease rates, there has been increased reliance on antimicrobials within aquaculture worldwide, especially in countries where regulatory limits lack clear definition and monitoring.

Antimicrobials refer to substances which include antibiotics, food additives, antiseptics, disinfectants, and other substances that act against microorganisms [2], disinfectants being substances that inhibit multiplication of microbes on inanimate/nonliving objects or surfaces. Substances such as iodophors, formalin, metal hydroxides and oxides, hydrogen peroxide, and potassium permanganate are used extensively in aquaculture facilities for control and prevention of fish diseases [1, 3, 4]. According to Manyi-Loh et al. [5], aiming to respond to the proliferation of pathogen and disease rates in aquaculture systems, farmers are quickly turning to the use of antimicrobials. Antimicrobials are used in fish farming to control bacterial diseases, ectoparasites, fungal outbreaks, as well as to improve poor water quality, sterilize eggs and equipment, and control aquatic weeds and free-living molluscs [6]. According to Austin and Austin [1], antimicrobials may be administered in fish through oral (via medicated feed), bath, dip, flush, topical application, or injection.

However, a seemingly gray area exists in the use and utilization of antimicrobial in aquaculture, tending to contradict what is observed on the ground. Available studies tend to suggest that there is low usage of antimicrobials in pisciculture in most developed countries, while in some countries, the usage appeared to decline with time. This has lead Manyi-Loh et al. [5] to postulate that the apparent scarcity of information on the amount of antibiotic used in aquaculture stems from the fact that only a few countries monitor the quantity of antibiotics utilized, since large quantities of antimicrobials are used in aquaculture in some countries often without professional consultation or supervision. In addition, as they are being used in land animal husbandry and human medicine, some antimicrobials may end up in water bodies with fish. These two factors may potentiate development of drug resistance exhibited by microorganisms. Manyi-Loh et al. [5] elaborate that antimicrobial resistance (AMR) is a phenomenon that predates antibiotic clinic usage, as it is an ancient process. Antimicrobial resistance in bacterial pathogens is a worldwide public health concern. This is of concern since, albeit the vast advancement being made in the aquaculture sector in Kenya, AMR continues to remain a gray area, due to there being no precise and concrete valuation on the quantity and existence of AMR in the Kenya aquaculture sector. This gap is a detrimental aspect for the fish veterinarian, pisciculturists, fisheries managers, policy planners, and fish farmers. There is, therefore, avid need to bridge this gap through effective and elaborate surveys and studies on AMR, so as to generate sufficient data for intervention purposes. This study was carried out to determine antibiotic and disinfectant susceptibility patterns of bacteria isolated from farmed fish in Kirinyaga County, Kenya.

## 2. Materials and Methods

### 2.1. Study Area and Design

The isolates used in the present study were recovered from apparently healthy farmed fishes (*Oreochromis niloticus*, *Clarias gariepinus*, *Carassius auratus*, and *Cyprinus carpio carpio*) and source pond water in Kirinyaga County, Kenya. The study was cross-sectional where bacteria were isolated and identified by conventional bacteriological methods following Markey et al. [7] and Austin and Austin [1] and by use of Analytic Profile Index 20E (API 20E) kits. Antibiotic and disinfectant susceptibility profiles of 48 bacterial isolates (belonging to the genera *Aeromonas*, *Proteus*, *Klebsiella*, *Citrobacter*, *Salmonella*, *Streptococcus*, *Pseudomonas*, *Escherichia*, *Serratia*, and *Micrococcus*) were established using the Kirby–Bauer disc diffusion method and agar well diffusion technique, respectively. Members of genera *Klebsiella, Salmonella, Escherichia*, and *Streptococcus* were tested due to zoonotic potential; members of the genus *Serratia*, *Micrococcus*, *Pseudomonas*, and *Aeromonas* were tested owing to their high pathogenicity potential in fish, as well as some being of public health concern, while members of the genus *Proteus* were tested due to their high prevalence. *Escherichia coli* (25922), *Pseudomonas aeruginosa* (27853), and *Staphylococcus aureus* (25923) from the American type culture collection (ATCC) were used as quality control organisms.

### 2.2. Antibiotic Susceptibility Testing

The antibiotic susceptibility patterns for the 48 bacterial pathogens were established in vitro, following the Kirby–Bauer disc diffusion method on Mueller–Hinton agar (Oxoid®), as described by Hudzicki [8] with certain modifications. There are no registered antibiotic formulations for use in aquaculture in Kenya, and therefore, the choice was guided by different classes of drugs reported elsewhere to treat diseases in aquaculture facilities [1]. Moreover, these drugs are equally used in veterinary and human medicine therapy. Eight commercially available antibiotic disks (Himedia^®^ OD 2/4) were used in the following concentrations: ampicillin (25 *μ*g); tetracycline (25 *μ*g); co-trimoxazole (25 *μ*g); streptomycin (10 *μ*g); kanamycin (30 *μ*g); gentamicin (10 *μ*g); sulphamethoxazole (200 *μ*g); and chloramphenicol (30 *μ*g).

The isolates and reference strains were inoculated on nutrient broth separately and incubated aerobically at 37°C. After overnight incubation, the bacterial suspension was vortexed and diluted to turbidity equivalent to that of 0.5 McFarland standards. The bacterial suspension was, then, spread onto the surface of the Mueller–Hinton agar (Oxoid) to make confluent growth. Antibiotic discs were immediately placed on the surface of the agar plate using forceps and incubated aerobically at 37°C for 16 hours. Inhibition zones for various isolates were measured and interpreted as sensitive, intermediate, or resistant according to the Clinical Laboratory Standards Institute (CLSI) [9–11].

### 2.3. Multiple Antibiotic Resistance Indices

The Multiple Antibiotic Resistance (MAR) indices of the study isolates against tested antibiotic were calculated using the formula MAR=*a*/*bc* , where “a” is the number of antibiotics to which the bacterial isolate was resistant, “b” is number of antibiotics tested, and “c” is the total number of isolates [12].

### 2.4. Disinfectant Susceptibility Testing

In order to determine the antibacterial profile of a disinfectant, the antibacterial susceptibility was performed by the agar well diffusion method on Mueller–Hinton agar as described by Schillinger and Lucke [14] and Njagi et al. [15], with certain modifications. Five (5) disinfectants available in the market were used at the concentration shown in Table 1.

Key: ^*∗*^User-recommended concentration by the manufacturer; ^*∗∗*^the concentrations are as recommended by Noga [16].

Owing to differences observed in phenotypic antibiotic susceptibility of isolates of the same genera, disinfectant analysis was carried out for all the 48 isolates against the 5 disinfectants. The bacteria suspensions were spread in the same way as for the antibiotic susceptibility testing. After spreading out, wells were punched using a sterile 6 mm diameter agar well-puncher. Dilutions of all the disinfectants used were prepared using sterile distilled water. Each disinfectant was dispensed separately, using a sterile micropipette, into different wells, using one Petri-dish per disinfectant. Each well was laden with 50 *μ*l of the pertinent disinfectant concentration. The Petri-dishes were then incubated in an upright position at 37°C. After 16 hours of incubation, each inhibition zone was measured and recorded in millimetres (mm). The results were construed as follows: a radius of 0–5 mm from the edge of the well to the inhibition front was taken as no inhibition; a radius of 6–9 mm was taken as a moderate inhibition; a radius of 10–14 mm was considered a strong inhibition, and beyond 15 mm was taken as a very strong inhibition [17].

### 2.5. Data Analysis

The collected data were validated, entered, and stored in a Microsoft Excel® spreadsheet, which was also used to calculate means and proportions. Descriptive and inferential statistics were performed using Statistical Package for the Social Sciences (SPSS®), version 22.0. The overall antibiotic response of each antibiotic was calculated as the number of bacteria resistant or sensitive to antibiotics over total number of bacteria isolates tested. One-way ANOVA was used to compute antibiotic and disinfectant susceptibility. All the tests were carried out at a significance level of 0.05.

## 3. Results

### 3.1. Antibiotic Susceptibility/Resistance Testing

Results of susceptibility testing for the 8 tested antibiotics on the 48 bacterial isolates are tabulated in Table 2. From the table, the overall susceptibility rates for each antibiotic for all the bacterial isolates were the highest in gentamicin (100%, *n* = 48) and kanamycin (92%, *n* = 44). Also, the bacterial isolates showed highest resistance rates to co-trimoxazole (73%, *n* = 34) and ampicillin (65%, *n* = 31). The overall antibiotic response for ampicillin, co-trimoxazole, streptomycin, kanamycin, sulphamethoxazole, and chloramphenicol was statistically significant (*p* < 0.05). However overall antibiotic response for tetracycline was insignificant (*p*=0.086).

**Figure 1:**
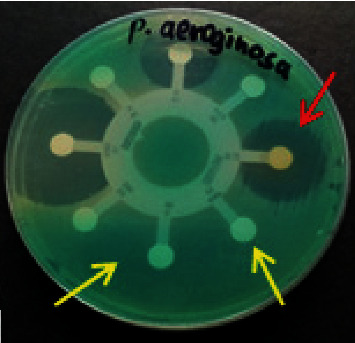
Antimicrobial susceptibility test results on the Mueller–Hinton agar culture plate showing susceptible (red arrow) and resistant (yellow arrow) zones of one of the isolates.

Figure 1 shows the antibiotic susceptibility pattern of one of the isolates. From the figure, there is a clear distinction between resistant (no inhibition) and susceptible (inhibition/clear zones).

### 3.2. Multiple Drug Resistance and Multiple Antibiotic Resistance Indices of the Isolates

Table 3 shows that the MAR indices of the bacterial isolates ranged from 0.1 to 0.8. The MAR index values of all the bacterial isolates were higher than 0.2 except *Micrococcus* spp. which had an MAR index of 0.1. This implies that most of the bacterial isolates were from high-risk source pollution such as manure-fertilized ponds. From the table, it can be seen that all the isolates showed multidrug resistant patterns to at least three antibiotics except *Micrococcus* spp., *Salmonella* enteritidis, and *Escherichia coli*. Genera *Proteus*, *Pseudomonas*, *Serratia*, and *Streptococcus* showed the highest MDR pattern, in that order.

### 3.3. Disinfectant Susceptibility Profiles of Bacterial Isolates

Table 4 gives the mean radius of the inhibition zone from the edge of the well to the inhibition zone front of disinfectants tested against selected bacteria. From Table 4, it can be gleamed that 3% and 1% hydrogen peroxide demonstrated the largest inhibition zone among the study bacteria at 23 mm to 20 mm zones, respectively, showing the broadest spectrum of antibacterial activity utilized by the study bacteria. Other disinfectants at different concentration(s) produced inhibition zones among the study bacteria as follows: by 2% formalin (19 mm), 1% iodine (15 mm), 1% formalin (14 mm), 0.5% iodine (12 mm), and 1.5% quaternary ammonium compound (10 mm).

**Figure 2:**
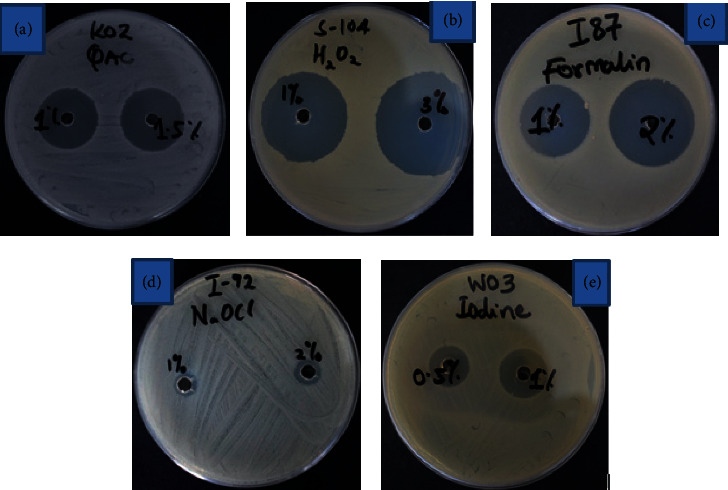
Antibacterial activity (clear areas) of various disinfectants to bacteria isolates (QAC (a), hydrogen peroxide (b), formalin (c), sodium hypochlorite (d), and iodine (e)).

Figure 2 shows disinfectant susceptibility patterns of study bacterial isolates against some disinfectants on Mueller–Hinton agar plates. The inhibition zone of the given disinfectant is shown by the clear areas.

Table 5 shows the extent of the inhibitory effect of the disinfectants tested on growth of test bacterial. Generally, most of the disinfectants showed an action of varying magnitudes against the bacterial isolates. The inhibitory activity of formalin and hydrogen peroxide at all concentrations was highly visible. The isolates were 100% sensitive to hydrogen peroxide and formalin at the recommended user-dilution. Iodine at user-recommended concentration showed moderate antibacterial activity. However, sodium hypochlorite at all concentrations showed little to no growth inhibition.

Inhibition zone interpretive criterion: - = No clear zone (0–5 mm); + = Moderate clear zone (6–9 mm); ++ = Strong clear zone (10–14 mm); and +++ = Very strong clear zone (≥ 15 mm).

## 4. Discussion

### 4.1. Antibiotic Resistance and Multiple Antibiotic Resistance Indexing

Antimicrobial resistance (AMR) is a worldwide public health concern that has drawn attention in the recent time. Existence of antibiotic resistance amongst these bacterial strains may have public health concern in fish consumers and handlers (fish harvesters and retailers). This is so especially if these resistant strains are transferred to humans. While some of these bacteria are ubiquitous to aquatic environments, some are exogenous or shed from the terrestrial environment into water bodies. Pond fertilization using livestock manure may also facilitate transfer of antibiotic-resistant bacteria from animals into aquatic life via soil and/or water [5]. This, therefore, calls for a holistic one health approach in curbing AMR scourge.

As corroborated by several other sources, AMR requires proper evaluation, monitoring, and assessment to articulate necessary legislative and regulatory measures [18]. As a potentially growing fishery nation, Kenya is yet to embark on formulating relevant regulations and legislations on the use of antibiotics aquaculture. It will be prudent to do so, so as to set ground for greater development in the Kenyan aquaculture sector similar to its international counterparts who are already on this path.

All the bacterial isolates showed multiple resistance to various drugs tested except for *Micrococcus* spp., *Salmonella enteritidis*, and *Escherichia coli*. However, all bacterial isolates were susceptible to gentamicin, and this is in agreement with the findings of Gufe et al. [13] and Karimi [19]. The resistance levels to kanamycin, streptomycin, chloramphenicol, and tetracycline were relatively low (below 20%). Indeed, cot-trimoxazole had highest levels of resistance; this in contrast with the findings of Newaj‐Fyzul et al. [20] who reported a relatively low resistance of 6.4%. Resistance against ampicillin was markedly high (65%); it is comparable to studies by Karimi [19] and Newaj‐Fyzul et al. [20].

Multiple antibiotic resistance indexing has been an effective and efficient tool in tracing bacteria exposure [21]. The MAR index values of all the bacterial isolates were higher than 0.2 except for the *Micrococcus* spp. which had an MAR index of 0.1. When the use of antibiotics is rare or underdosed for an animal treatment, the MAR value is equal to or less than 0.2. However, the MAR index value is greater than 0.2 in cases of high risk exposure with frequent therapeutic use of antibiotics in an animal. These findings further suggest the need to explore the role of pond fertilization using animal manure as a probable high-risk contaminant. Furthermore, the increased indiscriminate use and misuse of antibiotics (without veterinary supervision or consultation) in the livestock [22] and aquaculture sector is a major contributor to AMR and may have given rise to resistant bacteria. Since diseased fish are often treated as groups and not as individuals, this tends to expose both the healthy and sick fish in that population to the antimicrobials used.

### 4.2. Disinfectant Susceptibility Testing

Disinfection is applied as a common health management tool in aquaculture facilities [4], and the applications range from egg disinfection [23] to system disinfection [24]. Disinfectants are often used to inhibit fungal and bacterial growth and parasitic load in systems where preventive biosecurity measures are insufficient [25, 26], or it may be used for disease eradication (stamping out) efforts [4].

Precaution is crucial in the usage and dealing of disinfectants. The choice of disinfectant to use in an aquaculture establishment depends on the efficacy, volume required, cost, toxicity, and potential effluent concerns. The efficacy of liquid disinfectants is affected by the temperature, contact time, pH, concentration and presence of suspended solids, and organic and inorganic constituents [27].

Despite all the disinfectants tested being not registered and approved for use in aquaculture establishments in Kenya, results revealed the antibacterial potential of all 5 disinfectants tested. Among the disinfectants evaluated in this study, hydrogen peroxide and formalin were the most effective, with respect to the study bacteria, followed by iodine and quaternary ammonium compound (QAC). Hydrogen peroxide and formalin were effective at dilutions lower than what Noga [16] recommended while iodine and quaternary ammonium compound were only effective at the user-recommended concentration. Sodium hypochlorite showed slight to no effect at all the concentrations tested. This finding is in agreement with that of a study conducted by Njagi et al. [15] on sensitivity of the *Listeria* spp. recovered from indigenous chickens. Hydrogen peroxide (3%) showed the largest zone of inhibition, while sodium hypochlorite showed the smallest zone, with some isolates showing no inhibition (i.e., were resistant). Hydrogen peroxide does not affect the fish negatively and appears to improve water quality [28]. The use of hydrogen peroxide, especially in pond water disinfection, may be advantageous as its decomposition by-products (water and oxygen) are harmless and, therefore, environmental-friendly.

Formalin is one of the most used disinfectants in aquaculture to eliminate infectious agents; however, it may be responsible for adverse effects on fish and water quality [29]. Moreover, its carcinogenic potential to humans limits its use in fish intended for human consumption [30].

Quaternary ammonium compounds are promptly deactivated by organic matter and are usually ineffective against organisms such as *Pseudomonas*, *Proteus*, and other Gram-negative bacteria [27]. Chlorine compounds call for a contact time of 10–30 minutes to be potent and are highly corrosive and toxic [27].

## 5. Conclusions

Concomitant to other issues highlighted in this paper, the authors have demonstrated the existence of resistance among the fish bacterial isolates against antibiotics which might pose a challenge in disease control, as well as being hazardous to health of consumers and fish handlers. The authors have also demonstrated that all bacterial isolates were susceptible to most disinfectants tested. The potency of these disinfectants gives a reprieve in the wake of failing antibiotics.

### 5.1. Recommendations

To the fish veterinarians, managers, and farmers, it is recommended that, before making any therapeutic decision, antibiotic/disinfectant susceptibility testing is carried out, so as to use the effective antibiotic/disinfectant. There is also a need to strengthen government and industry institutions for aquatic animal health through development and enforcement of regulations on the use of antimicrobials. In addition, the institutions may consider registering and approving some of the chemical disinfectants for use in aquaculture establishments for the control of bacterial pathogens.

## Figures and Tables

**Table 1:** Disinfectants and their concentrations used in the disinfectants efficacy assay for the study bacteria.

Trade name	Chemical name	Active ingredient	Concentration
[1] Noro-cleanse® (Norbrook, Kenya)	Quaternary ammonium compound	15% glutaraldehyde and 10% coco-benzyl-dimethyl-ammonium chloride	1%
			1.5%^*∗*^

[3] Formalin	Formalin	40% formaldehyde	1%
			2%^*∗∗*^

[5] Hydrogen peroxide (Impact Chemicals, Kenya)	Hydrogen peroxide	6% hydrogen peroxide	1%
			3%^*∗∗*^

[7] Sodium hypochlorite solution (Diarim, Kenya)	Sodium hypochlorite	5% sodium hypochlorite	1%
			2%^*∗*^

[9] Novasept^®^(Navketan Research and Laboratories Pvt, India)	Iodine	1% available iodine	0.5%
			1%^*∗*^

**Table 2:** Antibiotic susceptibility patterns and overall antibiotic responses for the bacterial isolates, pegged on CLSI's inhibition zones interpretive criterion.

Bacterial isolates	Pattern	Number of isolates susceptible or resistant, *n* (%), to the antibiotic agents tested							
		AMP	TET	COT	S	K	GEN	SX	C
*A*. *hydrophila* (*N* = 6)	S	0 (0)	5 (83)	4 (67)	6 (100)	6 (100)	6 (100)	6 (100)	6 (100)
	R	6 (100)	1 (17)	2 (33)	0 (0)	0 (0)	0 (0)	0 (0)	0 (0)

*Proteus* spp. (*N* = 6)	S	0 (0)	2 (33)	0 (0)	6 (100)	6 (100)	6 (100)	3 (50)	3 (50)
	R	6 (100)	4 (67)	6 (100)	0 (0)	0 (0)	0 (0)	3 (50)	3 (50)

*K*. *pneuzmoniae* (*N* = 7)	S	6 (86)	5 (71)	5 (71)	7 (100)	7 (100)	7 (100)	7 (100)	7 (100)
	R	1 (14)	2 (29)	2 (29)	0 (0)	0 (0)	0 (0)	0 (0)	0 (0)

*C*. *freundii* (*N* = 6)	S	5 (83)	6 (100)	5 (83)	5 (83)	6 (100)	6 (100)	6 (100)	6 (100)
	R	1 (17)	0 (0)	1 (17)	1 (17)	0 (0)	0 (0)	0 (0)	0 (0)

*Salmonella enteritidis* (*N* = 4)	S	2 (50)	4 (100)	0 (0)	4 (100)	4 (100)	4 (100)	4 (100)	4 (100)
	R	2 (50)	0 (0)	4 (100)	0 (0)	0 (0)	0 (0)	0 (0)	0 (0)

*Streptococcus* spp. (*N* = 3)	S	0 (0)	2 (67)	0 (0)	0 (0)	1 (33)	3 (100)	1 (33)	3 (100)
	R	3 (100)	1 (33)	3 (100)	3 (100)	2 (67)	0 (0)	2 (67)	0 (0)

*P*. *aeruginosa* (*N* = 5)	S	0 (0)	4 (80)	0 (0)	5 (100)	5 (100)	5 (100)	0 (0)	0 (0)
	R	5 (100)	1 (20)	5 (100)	0 (0)	0 (0)	0 (0)	5 (100)	5 (100)

*E*. *coli* (*N* = 3)	S	2 (67)	3 (100)	0 (0)	3 (100)	3 (100)	3 (100)	3 (100)	3 (100)
	R	1 (33)	0 (0)	3 (100)	0 (0)	0 (0)	0 (0)	0 (0)	0 (0)

*S*. *marcescens* (*N* = 6)	S	0 (0)	6 (100)	0 (0)	4 (67)	4 (67)	6 (100)	4 (67)	6 (100)
	R	6 (100)	0 (0)	6 (100)	2 (33)	2 (33)	0 (0)	2 (33)	0 (0)

*Micrococcus* spp. (*N* = 2)	S	2 (100)	2 (100)	0 (0)	2 (100)	2 (100)	2 (100)	2 (100)	2 (100)
	R	0 (0)	0 (0)	2 (100)	0 (0)	0 (0)	0 (0)	0 (0)	0 (0)

Overall antibiotic response *(N* *=* *48)*	S	17 (35)	39 (81)	14 (27)	42 (88)	44 (92)	48 (100)	36 (75)	40 (83)
	R	31 (65)	9 (19)	34 (73)	6 (12)	4 (8)	0 (0)	12 (25)	8 (17)

*S: susceptible, R: resistance,* n: number of isolates, AMP: ampicillin, TET: tetracycline, COT: co-trimoxazole, S: streptomycin, K: kanamycin, GEN: gentamicin, SX: sulphamethoxazole, C: chloramphenicol.

**Table 3:** Multiple antibiotic resistance indices and multiple drug resistance patterns of bacterial isolates.

Bacterial strains	Antibiotic(s) to which the bacterial isolate was resistant to	MDR pattern	MAR index
*Micrococcus* spp.	COT	No	0.1
*Salmonella enteritidis*	AMP and COT	No	0.3
*Escherichia coli*	AMP and COT	No	0.3
*Aeromonas hydrophila*	AMP, TET, and COT	Yes	0.4
*Klebsiella pneumoniae*	AMP, TET, and COT	Yes	0.4
*Citrobacter freundii*	AMP, COT, and S	Yes	0.4
*Proteus* spp.	AMP, TET, COT, SX, and C	Yes	0.6
*Pseudomonas aeruginosa*	AMP, TET, COT, SX, and C	Yes	0.6
*Serratia marcescens*	AMP, COT, S, K, and SX	Yes	0.6
*Streptococcus* spp.	AMP, TET, COT, S, K, and SX	Yes	0.8

AMP: ampicillin, TET: tetracycline, COT: co-trimoxazole, S: streptomycin, K: kanamycin, GEN: gentamicin, SX: sulphamethoxazole, C: chloramphenicol, MDR: multiple drug resistance, MAR: multiple antibiotic resistance.

**Table 4:** Mean radius ± SD of the inhibition zone from the edge of the well to the inhibition zone front of disinfectants tested against the study bacteria.

Bacterial isolates	Sodium hypochlorite		Iodine		Hydrogen peroxide		Formalin		QAC	
	1%	2%^*∗*^	0.5%	1%^*∗*^	1%	3%^*∗∗*^	1%	2%^*∗∗*^	1%	1.5%^*∗*^
*Aer. hydrophila*	1 ± 1.0	2 ± 0.5	5 ± 0.5	8 ± 0.5	17 ± 0.5	21 ± 0.5	10 ± 0.5	14 ± 1.0	3 ± 0.5	5 ± 0.0
*Proteus* spp.	0 ± 0.0	0 ± 0.0	5 ± 0.0	8 ± 2.0	19 ± 0.5	23 ± 0.5	11 ± 0.5	15 ± 0.5	3 ± 1.0	5 ± 1.0
*Kleb. pneumoniae*	0 ± 0.0	0 ± 0.0	3 ± 0.0	6 ± 2.0	15 ± 0.0	18 ± 0.0	9 ± 1.0	14 ± 1.0	4 ± 0.5	5 ± 0.5
*Citr. freundii*	1 ± 0.0	3 ± 0.0	5 ± 1.5	9 ± 3.0	16 ± 1.5	19 ± 2.0	12 ± 0.5	15 ± 0.0	4 ± 0.5	6 ± 0.5
*Salm. enteritidis*	0 ± 0.0	0 ± 0.0	5 ± 0.5	10 ± 0.5	15 ± 1.0	20 ± 1.0	12 ± 0.5	14 ± 0.5	3 ± 0.0	5 ± 0.5
*Streptococcus* spp.	1 ± 0.5	2 ± 1.5	5 ± 1.0	7 ± 1.5	17 ± 3.0	21 ± 1.0	12 ± 2.0	15 ± 2.0	4 ± 2.0	6 ± 1.5
*Escherichia coli*	1 ± 0.0	2.0 ± 0	3 ± 0.0	5 ± 0.5	15 ± 0.5	20 ± 0.5	14 ± 0.5	19 ± 0.5	3 ± 0.5	6 ± 0.5
*Ser. marcescens*	1 ± 0.5	2 ± 1.5	4 ± 0.5	7 ± 0.5	12 ± 0.0	18 ± 1.0	11 ± 0.5	14 ± 1.5	2 ± 0.0	4 ± 0.0
*Ps. aeruginosa*.	0 ± 0.0	2 ± 0.5	3 ± 0.0	6 ± 0.5	13 ± 0.5	15 ± 1.0	9 ± 0.5	11 ± 0.5	4 ± 0.5	7 ± 0.5
*Micrococcus* spp.	0 ± 0.0	0.0 ± 0	10 ± 0.5	12 ± 1.0	11 ± 0.5	17 ± 0.5	14 ± 0.5	17 ± 0.5	9 ± 0.0	10 ± 0
Range	0–2	0–4	3–12	4–15	10–20	14–23	8–14	10–19	2–9	4–10
Mean	1 ± 0.2	1 ± 0.3	4.9 ± 0.6	7.9 ± 0.7	15 ± 0.6	19 ± 0.5	11 ± 0.4	15 ± 0.5	4 ± 0.4	6 ± 0.4

^*∗*^User-recommended concentration by the manufacturer; ^*∗∗*^the concentrations are as recommended by Noga [16]; QAC- quaternary ammonium compound; *Aer*.- *Aeromonas*; *Kleb*.- *Klebsiella*; *Citr*.- *Citrobacter*; *Salm*.- *Salmonella*; *Ser*.- *Serratia*; *Ps*. -*Pseudomonas*.

**Table 5:** Inhibitory activity of disinfectants on bacterial isolates.

Bacterial strains	Sodium hypochlorite		Iodine		Hydrogen peroxide		Formalin		QAC	
	1%	2%^*∗*^	0.5%	1%^*∗*^	1%	3%^*∗∗*^	1%	2%^*∗∗*^	1%	1.5%^*∗*^
*Aer. hydrophila*	_	_	_	+	+++	+++	++	++	_	_
*Proteus* spp.	_	_	+	+	+++	+++	++	+++	_	_
*Kleb. pneumoniae*	_	_	_	+	+++	+++	+	++	_	_
*Citrobacter freundii*	_	_	_	+	+++	+++	++	+++	_	+
*Salm. enteritidis*	_	_	_	++	+++	+++	++	++	_	_
*Streptococcus* spp.	_	_	_	+	+++	+++	++	+++	_	+
*Escherichia coli*	_	_	_	_	+++	+++	++	+++	_	+
*Ser. marcescens*	_	_	_	+	++	+++	++	++	_	_
*Ps. aeruginosa*	_	_	_	_	++	+++	+	++	_	++
*Micrococcus* spp.	_	_	+	++	++	+++	++	+++	+	++
Mean inhibition	_	_	_	+	++	+++	++	+++	_	+

^*∗*^User-recommended concentration by the manufacturer; ^*∗∗*^the concentrations are as recommended by Noga [16]; QAC- quaternary ammonium compound; *Aer*.- *Aeromonas*; *Kleb*.- *Klebsiella*; *Salm*.- *Salmonella*; *Ser*.- *Serratia*; *Ps*.- *Pseudomonas*.

## Data Availability

Data associated with this research article are available on request from the corresponding author.
